# Urgent actions to save lives when ICU bed needs approach or exceed capacity: lessons from Mongolia

**DOI:** 10.5365/wpsar.2022.14.5.942

**Published:** 2022-09-09

**Authors:** Buyantogtokh Batsukh, Bund-Ochir Khishigsaikhan, Dulamragchaa Buyanbaatar, Gerelmaa Danzan, Nansalmaa Munkhtur, Ariuntuya Ochirpurev, Takeshi Nishijima, Howard Sobel, Masahiro Zakoji

**Affiliations:** a Ministry of Health, Ulaanbaatar, Mongolia.; b World Health Organization Representative Office for Mongolia, Ulaanbaatar, Mongolia.; c World Health Organization Regional Office for the Western Pacific, Manila, Philippines.

**Fig. 1:**
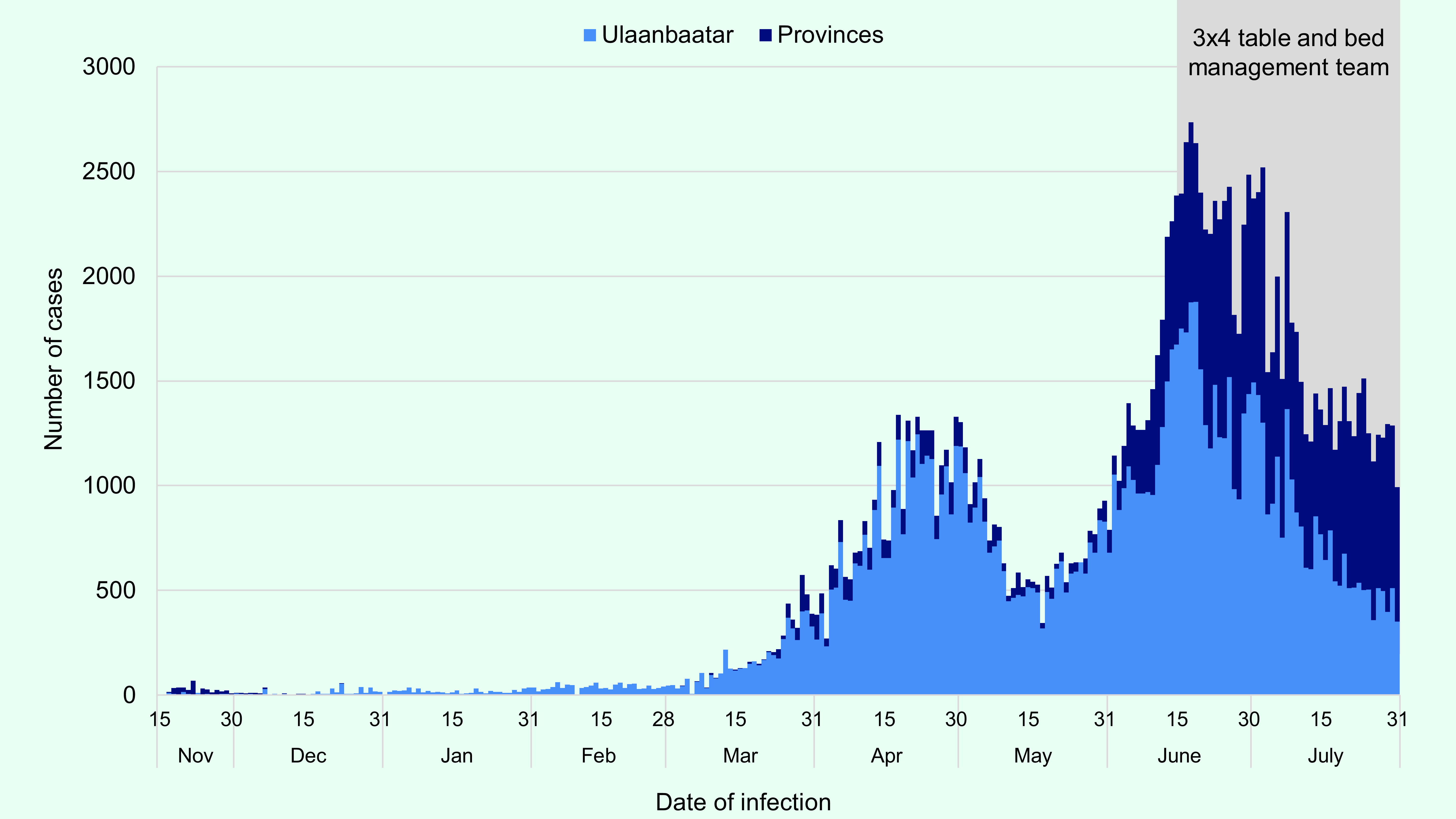
Reported daily cases of COVID-19 by Ulaanbaatar and provinces, Mongolia, November 2020–July 2021

**Fig. 2:**
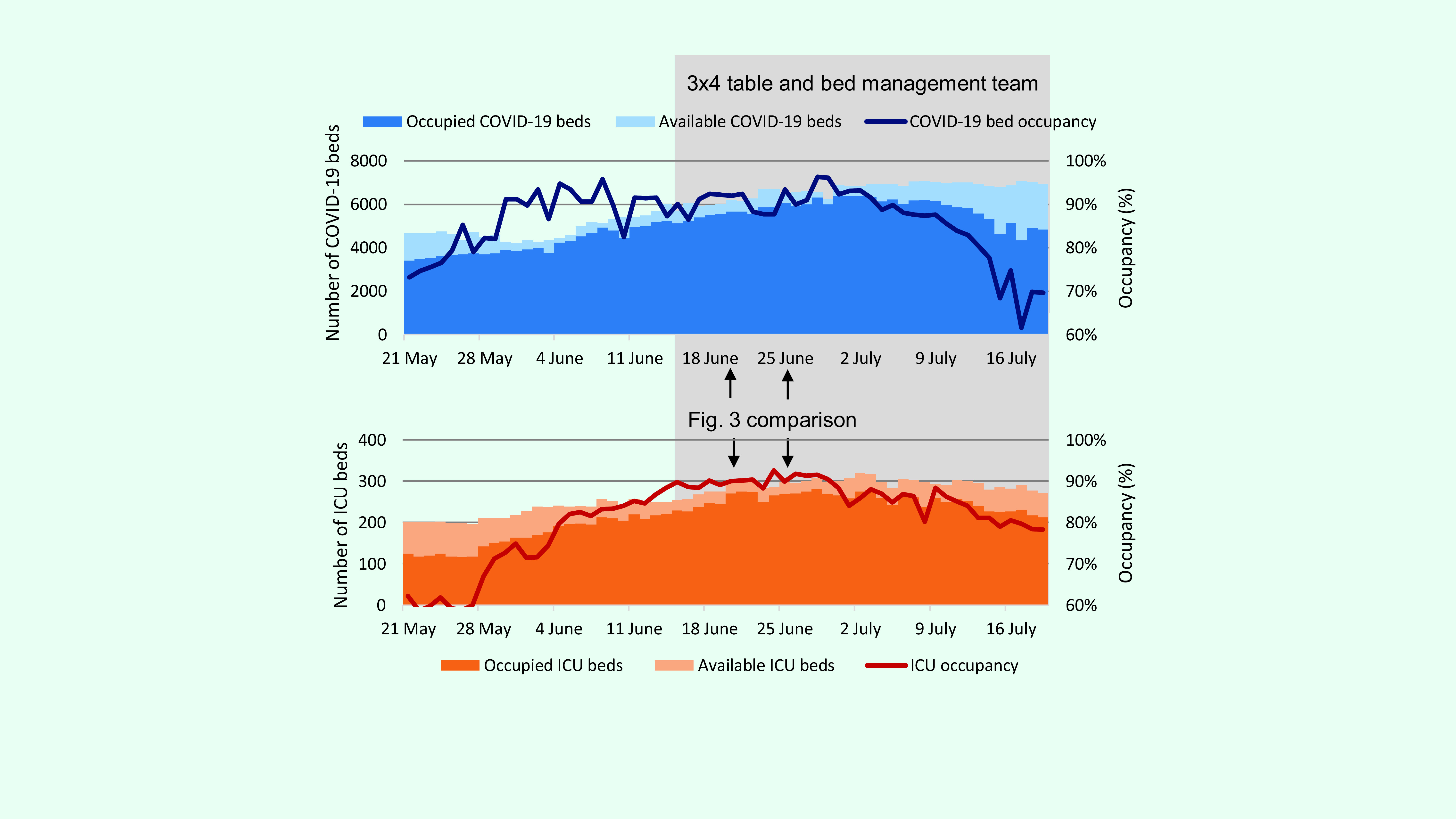
COVID-19 bed and ICU occupancy in Ulaanbaatar, 21 May–19 July 2021

At the beginning of the coronavirus disease 2019 (COVID-19) pandemic, Mongolia took early and stringent response measures that were considered successful until early 2021. (*1*, *2*) Following the lifting of a nationwide lockdown in April 2021, there was a rapid resurgence of cases from mid-May to mid-June (**Fig. 1**). By early June, COVID-19 hospital bed and intensive care unit (ICU) bed occupancy in the capital of Ulaanbaatar exceeded total capacity (**Fig. 2**). This impacted both health-care delivery for COVID-19 and other essential health services. At its peak, 2746 new cases (18 June 2021) and 17 deaths (3 July 2021) were reported in a single day, totalling 166 145 cases and 812 deaths as of 1 August 2021. (*3*)

[**insert** Figure 1]

[**insert** Figure 2]

Mongolia is a lower middle-income country with a population of 3.3 million widely distributed across a vast area of over 1.5 million km^2^. Health service delivery is organized into national, provincial and subprovincial levels. There is an average of 80 beds and 30 medical doctors per 10 000 population, with higher ratios in Ulaanbaatar than in the provinces. (*4*)

World Health Organization (WHO) clinical management guidelines recommend that COVID-19 care pathways be established at the national, subnational and local levels to treat patients in the right settings according to disease severity and risk. (*5*) However, the national distribution of COVID-19 patients of different disease severity across the health system has rarely been systematically monitored or documented in Mongolia.

In response to the increasingly overwhelmed health capacity, the Ministry of Health and WHO conducted a rapid systems assessment and took action on three key components: influx of patients, care pathway and exit. To manage the influx of new patients into care pathways, more stringent public health and social measures (PHSMs) such as restrictions on business operation and interprovincial movement were introduced from mid-June 2021. To increase care capacity, 1947 additional beds were mobilized by mid-June including approximately 100 additional ICU beds and newly established intermediate facilities and treatment centres in Ulaanbaatar. Intermediate facilities with oxygen supplies and temporary ICU beds accommodated primarily non-severe patients with risk factors for severe disease and severe patients who needed oxygen, while treatment centres provided care for severe and critical patients. Severe patients in intermediate facilities were referred to treatment centres as bed availability and their condition allowed. Despite these measures, bed occupancy was rapidly overwhelmed. By 14 June, 33 deaths were reported among patients with severe disease monitored at home who rapidly deteriorated.

**Fig. 3:**
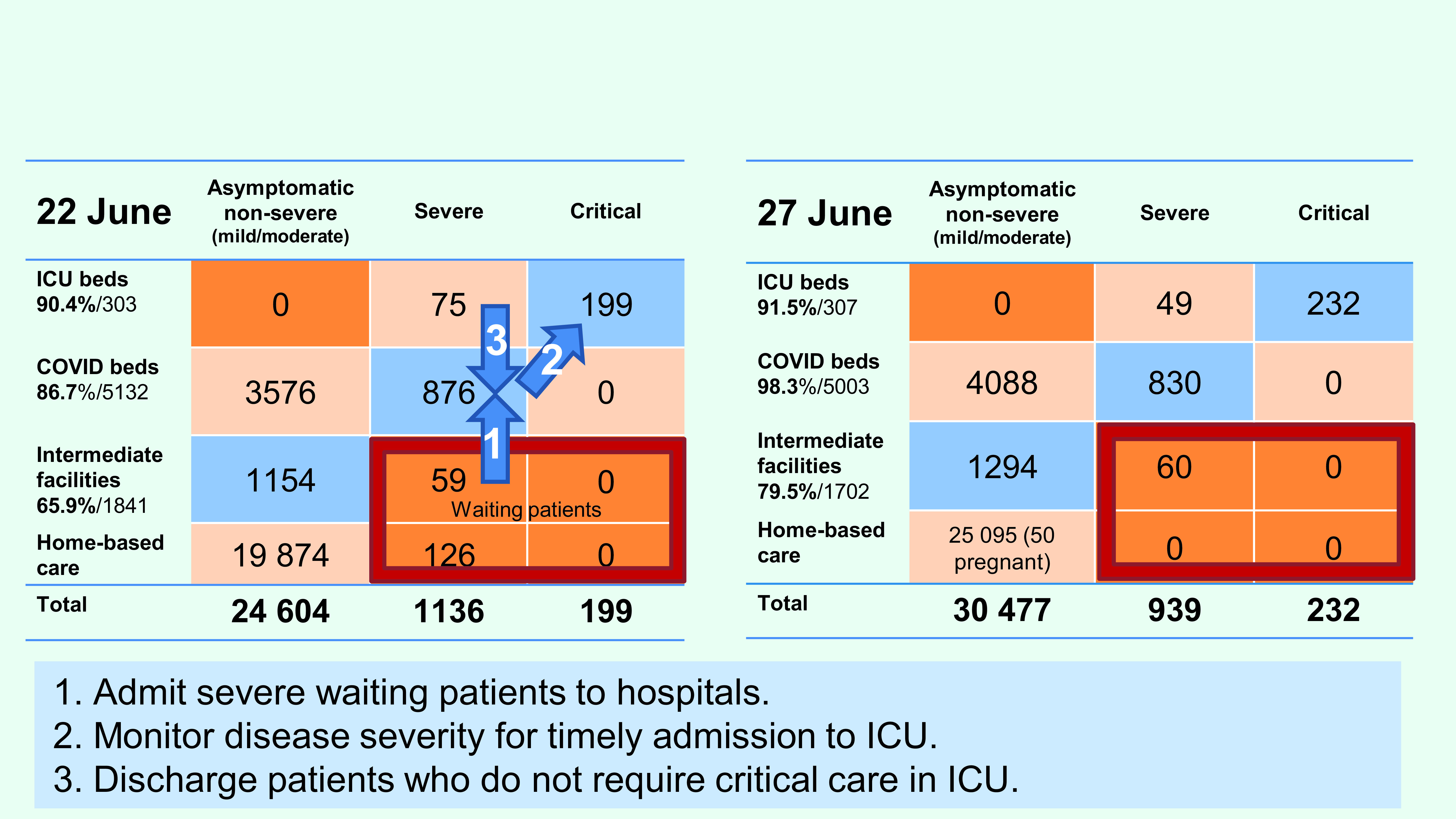
Patient distribution by severity and facility for two time periods, 22 and 27 June 2021, Ulaanbaatar, Mongolia

WHO supported the Ministry of Health to map cases into a 3x4 table by disease severity and type of facility as per WHO clinical management guidance in Ulaanbaatar and provinces (**Fig. 3**). (*5*) Numbers of available beds and patients were reported by each health facility and collated on an online dashboard. A bed management team, comprised of seven members from the Ministry of Health, National Center for Communicable Diseases and the City Health Department, was established on 17 June to oversee health-care utilization at different levels of the health system and coordinate admissions and referrals to optimize the use of resources. By assessing the table from highest to lowest disease severity, three urgent actions were identified, agreed upon and implemented within 2 weeks.

[**insert** Figure 3]

First, all patients with severe disease who were at home were admitted. As of 22 June, the 3x4 table analysis identified 126 patients with severe disease who were at home waiting for hospitalization. Family doctors and district surveillance doctors were monitoring the severity of patients at home via in-person visits or over the phone. Between 22 and 27 June, all of these patients were hospitalized or kept at the newly built intermediate triage and treatment centre, which was equipped with temporary critical care resources including mechanical ventilators.

Second, patients with severe disease or risk factors for severe disease who were in non-ICU COVID-19 beds in hospitals and intermediate care facilities were closely monitored using pulse oximetry for timely admission to the ICU. While severe cases in general wards decreased from 876 to 830 between 22 and 27 June, 33 patients requiring critical care were identified and moved to the ICU.

Third, to create space in the ICU, patients who did not require intensive care were discharged. ICU patients were reassessed daily for disease severity and were discharged to COVID-19 general wards when appropriate. Of the 75 severe patients occupying ICU beds who did not require mechanical ventilation or vasopressor therapy, 26 were transferred to general wards. This increased efficiency in allocating limited critical care resources to patients who most needed them.

In the period following these actions, deaths decreased from a peak of 104 during the week of 28 June to 41 during the week of 19 July and further decreased thereafter. Through live monitoring of bed occupancy, the COVID-19 care pathway continued to be proactively fine-tuned after this initial phase.

By improving the efficient use of COVID-19 and ICU beds, space was made for patients with severe disease or risk factors for severe disease where monitoring was more intense and referral easier. This resulted in immediate reduction of waiting patients. Accomplishing this required that a strict definition of disease severity and corresponding care be ensured and applied, such as that in the WHO clinical management guidelines.

Fear of deterioration both among the public and clinicians, coupled with a financial incentive for hospitals to admit mild cases, were the main drivers behind inefficient bed management. Assuring safe home monitoring and timely admission and updating the reimbursement policy to require approval from bed management teams helped manage conflicting expectations and interests.

When service capacity is near or exceeding the maximum, urgent actions must be taken to minimize preventable deaths. (*6*) Clinical care pathways alone cannot solve the issue; a comprehensive systems approach, including PHSMs, point-of-entry measures and vaccination, is critical to augment severity-based efficient bed management. The 3x4 table mapping is a simple yet powerful framework to visualize the distribution of patients at different levels across the health system and help policy-makers and facility managers take urgent decisions to save lives.

The limitations of this approach include the possible misclassification of disease severity, data inadequacy and lateness, and the additional workload of monitoring in a disaggregated manner. It is also not possible to conclude if and to what extent the improved bed management contributed to minimizing preventable deaths.

To safeguard against surges overwhelming health systems and ensuring care for the right patients in the right settings, the hospital-centred COVID-19 care pathway needs to be adapted to be more comprehensive, integrating home and intermediate facilities. To that end, safe monitoring, timely referral and optimized bed management are key. For sustained management of COVID-19, it is critical to strengthen multisource surveillance as described in the Asia Pacific Strategy for Emerging Diseases and Public Health Emergencies (APSED III), including health-care capacity to inform proactive policy decisions and adaptations to health-care pathways. (*7*)
